# NCAPG promotes the oncogenesis and progression of non-small cell lung cancer cells through upregulating LGALS1 expression

**DOI:** 10.1186/s12943-022-01533-9

**Published:** 2022-02-18

**Authors:** Huanhuan Sun, Hong Zhang, Yan Yan, Yushi Li, Gang Che, Cuiling Zhou, Christophe Nicot, Haiqing Ma

**Affiliations:** 1 Medical Research Center, Guangdong Provincial People’s Hospital, Guangdong Academy of Medical Sciences, 106 Zhongshan Er Rd, Guangzhou, 510080 Guangdong China; 2 Department of Oncology, The Fifth Affiliated Hospital, Sun Yat-sen University, Zhuhai, China; 3 Department of Oncology, Guangdong Cardiovascular Institute, Guangdong Provincial People’s Hospital, Guangdong Academy of Medical Sciences, Guangzhou, China; 4 Department of Pathology and Laboratory Medicine, University of Kansas Medical Center, 3901 Rainbow Boulevard, Kansas City, KS 66160 USA

**Keywords:** NSCLC, NCAPG, LGALS1, Oncogenesis, Progression

## Abstract

**Background:**

Numerous common oncogenic driver events have been confirmed in non-small cell lung cancer (NSCLC). Although targeted therapy has revolutionized NSCLC treatment, some patients still do not respond. NCAPG, also known as non-SMC condensin I complex subunit G, was positively associated with proliferation and migration in several tumor types.

**Methods:**

We used transcriptional sequencing and TCGA database analysis to identify *NCAPG* as a new therapeutic target for NSCLC. The oncogenic roles of NCAPG in NSCLC tumor growth and metastasis were detected in vitro and in vivo. *Ncapg*^*+/+*^ or *Ncapg*^*+/−*^ mice with urethane treatment were analyzed for oncogenesis of NSCLC.

**Results:**

We investigated *NCAPG* as a new oncogenic driver which promoted NSCLC tumorigenesis and progression. We used transcriptome sequencing and the Cancer Genome Atlas (TCGA) database analysis to screen and found that *NCAPG* was negatively correlated with NSCLC survival. Using immunohistochemistry, we demonstrated that NCAPG overexpression was an independent risk factor for NSCLC survival. Functionally, *NCAPG* knockdown inhibited proliferation, migration, and invasion of NSCLC cells in vitro and in vivo. We exposed wildtype or *Ncapg*^*+/−*^ mice to urethane and discovered that urethane-induced lung tumors were reduced in *Ncapg*^*+/−*^ mice. Mechanistically, the function of NCAPG in promoting initiation and progression of NSCLC was closely related to LGALS1, which was also upregulated in NSCLC and might interact directly with NCAPG.

**Conclusions:**

This study indicates that *NCAPG* is one of the essential factors for NSCLC oncogenesis and progression, providing a new target for prognosis prediction and treatment of NSCLC.

**Supplementary Information:**

The online version contains supplementary material available at 10.1186/s12943-022-01533-9.

## Introduction

The discovery of actionable oncogenic drivers includes epidermal growth factor receptor (EGFR) [1], anaplastic lymphoma kinase (ALK) [2], mesenchymal-epithelial transition factor (MET) [3], Kirsten rat sarcoma 2 viral oncogene homolog (KRAS) [4], serine/threonine-protein kinase b-raf (BRAF) [5], and ROS1 proto-oncogene receptor tyrosine kinase (ROS1) [6], which have revolutionized the molecular targeted therapy of NSCLC. However, these specially targeted inhibitors only provide clinical benefits to patients with related driver gene mutations, and these patients still have a challenge with acquired drug resistance. Discovering new potential biomarkers is urgently needed for NSCLC treatment.

To identify new therapeutic targets for NSCLC, we used transcriptional sequencing and TCGA database analysis and found that NCAPG might be involved in NSCLC development regulation. The non-SMC subunits are responsible for ATP-dependent DNA supercoiling and chromosome segregation [7]. NCAPG, also known as non-SMC condensin I complex subunit G, was first isolated from HeLa cell nuclei and demonstrated to regulate the location of DNA on chromosomes [8]. Oncogenic NCAPG is positively associated with S phase cell cycle arrest, proliferation and migration in several tumor types, including gastric cancer [9], breast cancer [10], hepatocellular carcinoma (HCC) [11], and etc. Meanwhile, the clinical importance of NCAPG’s biology is that its overexpression was associated with poor prognosis in HCC [12] and renal cell carcinoma [13]. However, the role of NCAPG in NSCLC and its related mechanisms require elucidation.

Here, we demonstrated the ability of NCAPG to promote the proliferation, migration, invasion, and metastasis in NSCLC. By analyzing the Cancer Genome Atlas (TCGA) database and clinical samples of NSCLC, we found that NCAPG was the only differentially expressed gene that negatively correlated with the survival of NSCLC patients. Additionally, we investigated the function of NCAPG on tumorigenesis of NSCLC by knocking it out in a murine lung cancer model. Furthermore, LGALS1 was identified to be interacted with NCAPG to participate in NSCLC progression. As a result, we hypothesized that *NCAPG* could be a potential biomarker and therapeutic target in NSCLC.

## Materials and methods

### TCGA and GEO database analyses

Between 1991 and 2013, data on 999 adenocarcinoma and squamous cell carcinoma NSCLC cases were extracted from the TCGA database (https://cancergenome.nih.gov/). These data were used to analyze differential expression, correlation, and patient survival. Table S1 contains clinical data on the 999 cases. The data of GSE102287 were obtained from Gene Expression Omnibus (GEO) database (https://www.ncbi.nlm.nih.gov/gds/). These data were analyzed by differential expression analysis.

### Cell lines and cultures

NSCLC cell lines including HCC827, H460, H1299, H1975, A549, and PC9 were purchased from the American Type Culture Collection (ATCC). H460, H1299, H1975, and HCC827 were cultured in ATCC-formulated RPMI-1640 medium. PC9 cells were cultured in high-glucose Dulbecco’s modified eagle medium (DMEM). A549 cells were cultured in an ATCC formulated F-12 K medium. Each medium contained 10% FBS and 100 U/mL penicillin. These cells were cultured at 37 °C and in a humidified incubator at 5% CO2.

### NSCLC specimens

Between February 2010 and November 2014, 156 pairs of NSCLC tumor and adjacent normal tissue specimens were obtained from patients who underwent surgeries at the Fifth Affiliated Hospital of Sun Yat-sen University and Sun Yat-sen University Cancer Center. Table 2 lists the clinicopathological characteristics of 156 patients. None of these patients received radiotherapy or chemotherapy before surgery, and all received standard therapy following surgery. The histologic types were determined following the criteria set by the World Health Organization (WHO). All procedures followed were in accordance with the ethical standards of the responsible committee on human experimentation (institutional and national) and the Helsinki Declaration of 1975, as revised in 2000. All patients provided informed consent to participate in the study.

### Proximity ligation assays (PLA)

PLA was carried out with Duolink Detection Kit (Sigma, DUO92101-1KTT) according to manufacturer’s instructions. Briefly, fixed and permeabilized cells were incubated for 30 min at 37 °C in a blocking solution. The cells were incubated with primary antibodies diluted in Duolink antibody diluents overnight at 4 °C. After washes, the cells were incubated for 60 min at 37 °C with appropriate PLA probes. Following washes, circularization and ligation of appropriate oligonucleotides were performed in ligase-containing solution for 30 min at 37 °C. The cells were then rinsed briefly and incubated for 100 min at 37 °C with an amplification solution. After PBS washes, coverslips were mounted with Duolink in Situ Mounting Medium with DAPI. Samples were analyzed using a laser-scanning confocal microscope and multispectral imaging flow cytometry. These experiments were repeated at least three times.

### 5-ethynyl-2′-deoxyuridine assay

A549 and H1299 cells were transfected with NCAPG shRNA and/or LGALS1 overexpression vectors. And the cells were cultured in medium contained 5-ethynyl-2′-deoxyuridine (EdU) (30 μM) (Cell Light EdU DNA imaging Kit, Guangzhou RiboBio, China) for 2 h. The levels of EdU were detected by flow cytometry according to the manufacturer's protocol.

### Animal experiments

All animals were purchased from Sun Yat-sen University Animal Center. All animal experiments were approved by the Ethics Committees of Guangdong Provincial People’s Hospital and the Fifth Affiliated Hospital of Sun Yat-sen University. This study was performed following the ethical standards of the Declaration of Helsinki. Additionally, all institutional and national guidelines for the care and use of laboratory animals were followed. To evaluate the role of NCAPG in the growth of NSCLC, 20 BALB/c nude female mice (8 weeks old) were randomly divided into two groups (*n* = 10 per group). A549 (5 × 10^6^) and H1299 (2 × 10^6^) cells transfected with either LV-shNCAPG or LV-shCtrl were injected subcutaneously into each axilla of mice separately. Tumor volumes were measured every four days and calculated with the formula: volume = 0.5 × length × width^2^. The mice were sacrificed after 4–5 weeks, and the tumors were weighed.

For the in vivo pulmonary metastasis murine model, ten NOD/SCID mice (6 weeks old) were injected through the tail vein with H1299 (1 × 10^6^) cells transfected with either LV-shNCAPG or LV-shCtrl. The mice were sacrificed after 31 days and the lungs were harvested and analyzed for the presence of metastatic tumors.

Heterozygous *Ncapg* mice were generated by Cyagen Biosciences Inc. (Suzhou, China) in order to intercross for homozygotes. Exons 2, 3, 4 and 5 of mouse *Ncapg* were knockout by the CRISPR Cas9 system. The following mouse *Ncapg* sgRNA sequences were used: sgRNA F: GTGTGTCGAGTGTAGCGGCGGGG; sgRNA R: GAGAGGGTGGGGCATTATACTGG. Genetic deletion analysis for *Ncapg* was examined by multiplex genomic DNA PCR according to the manufacturer’s protocol (Cyagen Biosciences Inc). Primer sequences were as follows: F1: 5′-AGTGACTTGTTGATCTGCGCAG-3′; R1: 5′-GCCAAACTCCTACATCACCTATG-3′; R2: 5′-CTCTCAACGCCTTTGGATTATCG-3′. However, no homozygous *Ncapg* knockout mice have ever been born. A urethane-induced lung tumor model was used to investigate the role of *Ncapg* in NSCLC initiation. A total of 20 *Ncapg*^+/−^ mice and 20 *Ncapg*^+/+^ C57BL/6 mice were intraperitoneally injected with urethane (1.0 mg/g body weight) once per week for six weeks. The mice were sacrificed 12 weeks after urethane treatment, and the lungs were harvested and further analyzed. Genotype results of mouse embryonic fibroblasts (MEFs) from offspring mice embryos are provided in Table S2.

### Immunoprecipitation-MS

Previous studies demonstrated the use of immunoprecipitation-MS to detect NCAPG- interacting proteins [14, 15].

### Statistical analysis

A paired-samples *t*-test was used to compare mRNA and protein expression of *NCAPG* and *LGALS1* in NSCLC tumors and the paired adjacent normal tissue samples. The *χ*^2^ test was used to analyze the relationship between NCAPG expression and various clinicopathologic characteristics. The Kaplan-Meier method was used to calculate the survival curves, and the log-rank test was used to compare them. For univariate and multivariate analyses, Cox proportional hazard regression model was used to determine the effect of clinicopathologic variables and NCAPG expression on survival. All statistical analyses were conducted using SPSS 16.0 software (SPSS, Chicago, IL), and a *p*-value < 0.05 was considered statistically significant [16]. In addition, the remaining methods are presented in Supplementary Methods, Figures and Tables*.*

## Results

### NCAPG is upregulated in NSCLC

**Fig. 1:**
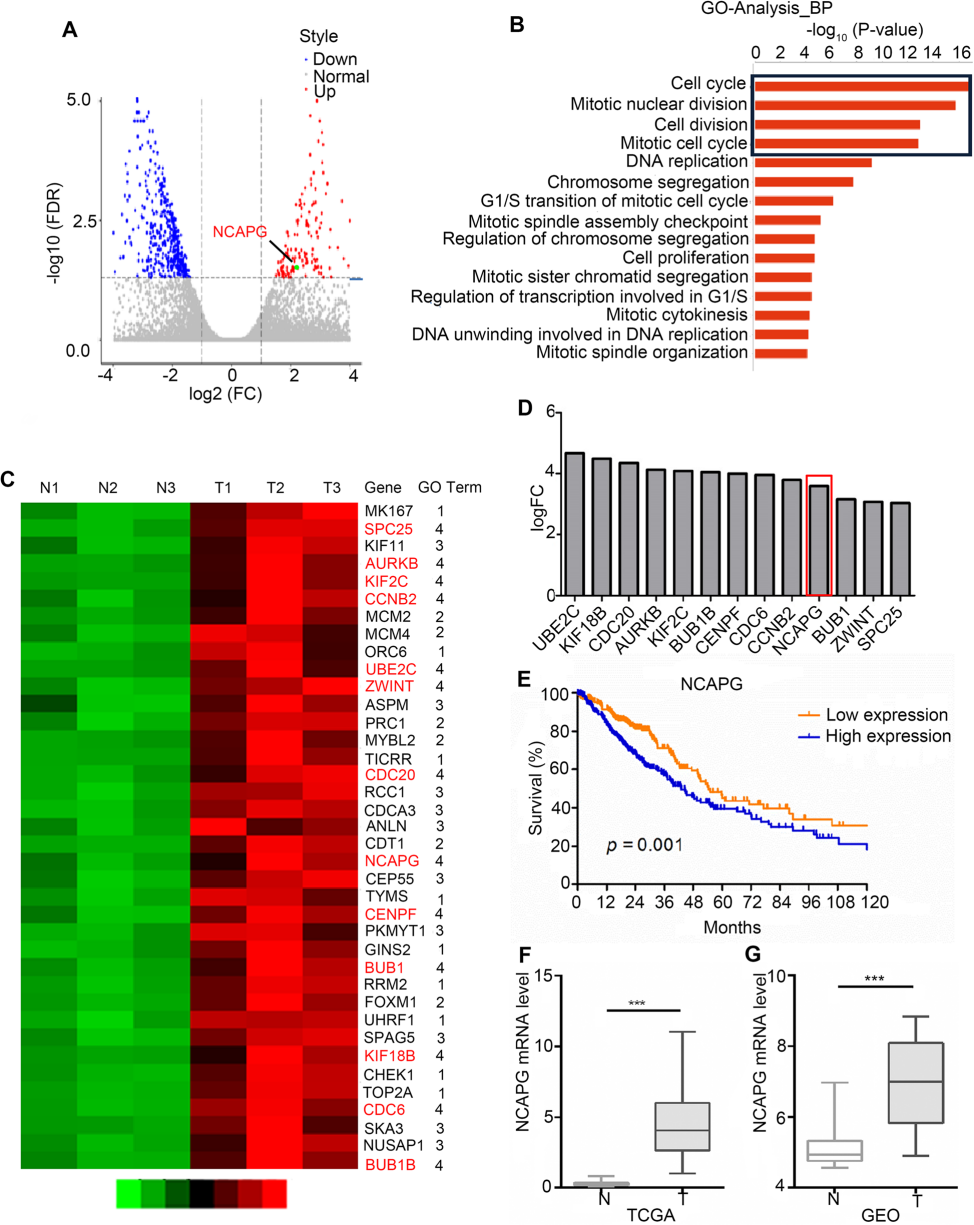
Identification of NCAPG expression in NSCLC. **A** Gene sequencing volcano plots of NSCLC tumor tissues and adjacent normal tissues (blue indicates downregulated genes; red indicates upregulated genes). **B** GO-BP analysis of upregulated genes in tumor tissues. **C** Heatmaps of all 39 upregulated genes involved in cell cycle, mitotic division, cell division, and mitotic cell cycle. **D** The 13 upregulated genes highly expressed in NSCLC tumor tissues from the TCGA database. **E** The relationship between NCAPG expression and survival in NSCLC from the TCGA database. **F**, **G** *NCAPG* expression level in the TCGA and GEO databases. (*** *p* < 0.001)

To identify new therapeutic targets of NSCLC, we performed transcriptome sequencing on three assembled paired tumor tissue samples and adjacent normal tissues. We identified 415 downregulated genes and 158 upregulated genes (Fig. 1A, log2FC > 1 or < − 1, false discovery rate (FDR) <  0.05). GO analysis revealed that 39 upregulated genes were frequently involved in four biological processes including cell cycle, mitotic nuclear division, cell division, and mitotic cell cycle (Fig. 1B, C). Surprisingly, 13 genes were simultaneously involved in these four biological processes (Fig. 1C). Moreover, TCGA data analysis showed that the 13 genes were overexpressed in NSCLC tumor tissues (Fig. 1D, log2FC > 2, *p <* 0.001), but only *NCAPG* was significantly correlated with NSCLC patient survival (Fig. 1E, Fig. S1, *n* = 500, high expression cases = 250, low expression cases = 250, *p* = 0.001). Consistent with TCGA data analysis results (Fig. 1F), GEO data analysis also showed that NCAPG were highly expressed in NSCLC tumor tissues (Fig. 1G, *p <* 0.001). Furthermore, *NCAPG* expression was significantly correlated with TNM stage (*n* = 970, *p <* 0.001), T stage (*n* = 970, *p <* 0.001), and N stage (*n* = 970, *p <* 0.001) in NSCLC patients (Table 1). The above results suggest that *NCAPG* expression is positively related to NSCLC.

**Table 1:** NSCLC stage and their correlation with *NCAPG* expression in TCGA database

Characteristic	NCAPG expression		*p*-value	r
	Negative n (%)	Positive n (%)		
Tumor stage				
pT1	175 (63.6)	100 (36.4)	< 0.001	0.17
pT2	249 (45.9)	294 (54.1)		
pT3	44 (39.3)	68 (60.7)		
pT4	17 (42.5)	23 (57.5)		
Node metastasis				
pN0	343 (54.2)	290 (45.8)	< 0.001	0.127
pN1/ pN2/pN3	142 (42.1)	195 (57.9)		
TNM stage				
I	290 (58.0)	210 (42.0)	< 0.001	0.198
II	115 (41.4)	163 (58.6)		
III	67 (40.9)	97 (59.1)		
IV	13 (46.4)	15 (53.6)		

**Fig. 2:**
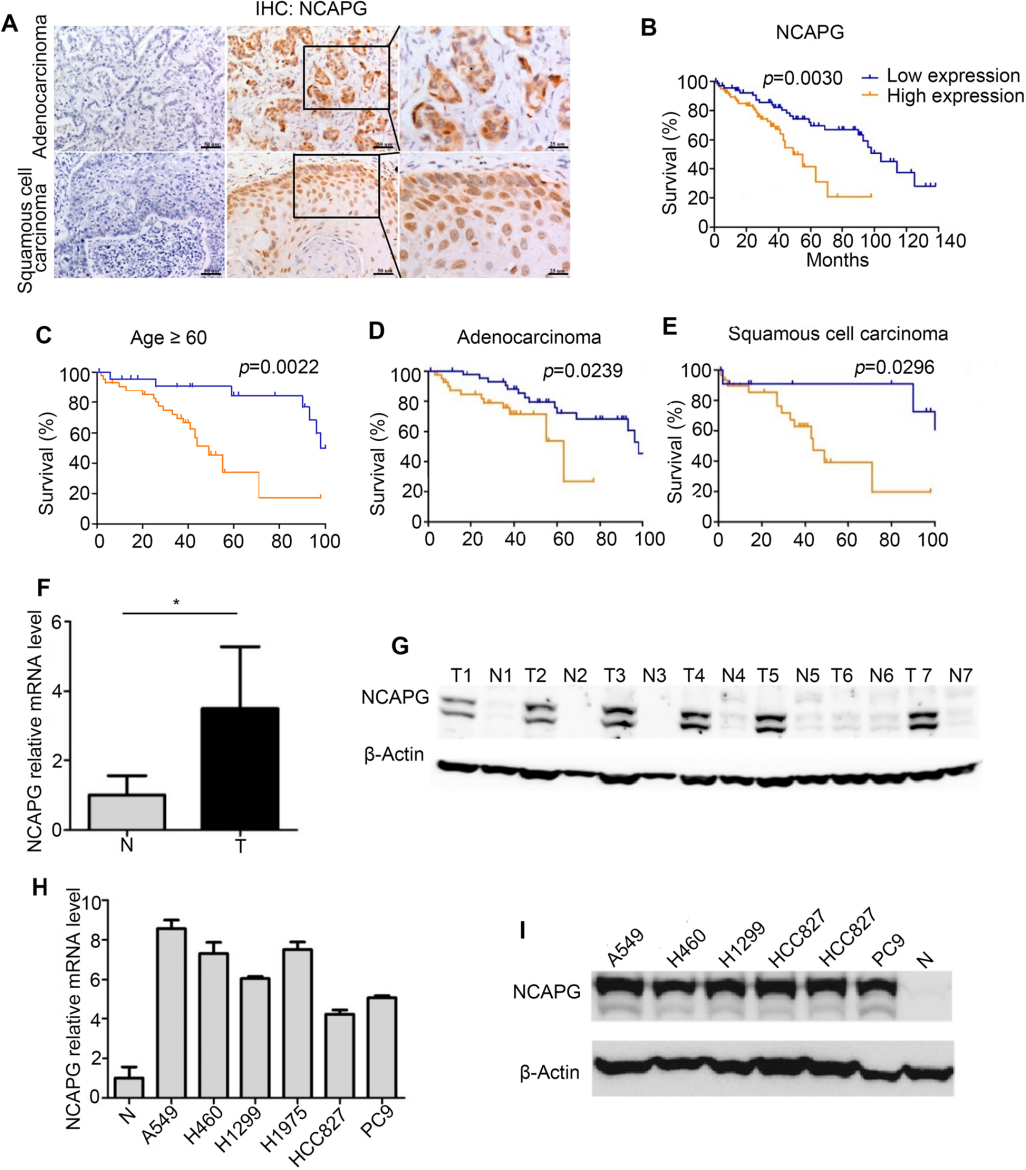
Overexpression of *NCAPG* in NSCLC tissues and cell lines. **A** NCAPG expression in NSCLC tumor tissues by IHC. Left: negative; middle & right: positive; top: adenocarcinoma; bottom: squamous cell carcinoma. **B** The relationship between NCAPG expression and survival in NSCLC patients. **C-E** This negative correlation between NCAPG expression and survival was identified in elderly patients (≥ 60 years old) (*n* = 84, *p* = 0.0022), adenocarcinoma (*n* = 92, *p* = 0.0239), and squamous cell carcinoma (*n* = 43, *p* = 0.0296). **F**, **G** *NCAPG* mRNA (**F**) and protein expression (**G**) in NSCLC tumor tissues (*n* =21) (T: tumor tissues, N: matched adjacent normal tissues). **H**, **I** *NCAPG* mRNA (N: Average *NCAPG* mRNA expression in adjacent normal tissues (*n* = 21)) and protein (N: Representative protein level of *NCAPG* in adjacent normal tissues) expression in NSCLC cell lines

To further investigate the expression and prognostic value of *NCAPG*, IHC was performed to assess NCAPG expression in NSCLC tumor tissues (Fig. 2A). Our study demonstrated that the expression level of NCAPG was correlated with gender, age, and histopathologic type (Table 2). Kaplan-Meier survival analysis showed that NCAPG expression was negatively correlated with survival in NSCLC patients (Fig. 2B, *n* = 156, *p* = 0.003), particularly in elderly patients (≥ 60 years old) (Fig. 2C, *n* = 84, *p* = 0.0022), adenocarcinoma (Fig. 2D, *n* = 92, *p* = 0.0239), and squamous cell carcinoma (Fig. 2E, *n* = 43, *p* = 0.0296). Cox regression analysis further demonstrated that NCAPG expression was an independent risk factor for NSCLC patient survival (Table 3, *p* = 0.022). Importantly, both RT-qPCR and Western blot analyses showed that *NCAPG* expression was significantly increased in tumor tissues when compared with adjacent normal tissues (Fig. 2F, G). In addition, NCAPG was notably overexpressed in six NSCLC cell lines, with the highest expression in A549 cells (Fig. 2H, I).

**Table 2:** Patients’ characteristics and their association with NCAPG expression

Characteristic NCAPG expression	Negative (*N* = 72),	Positive (*N* = 84),	*P* -value
	n (%)	n (%)	
Gender			0.014
Male	34 (38)	56 (62)	
Female	38 (58)	28 (42)	
Age			0.020
< 60	46 (55)	38 (45)	
≥ 60	26 (36)	46 (64)	
Tumor stage			0.523
pT1	12 (39)	19 (61)	
pT2	38 (53)	34 (47)	
pT3	14 (56)	11 (44)	
pT4	2 (50)	2 (50)	
Node metastasis			0.235
pN0	47 (54)	40 (46)	
pN1/ pN2	18 (43)	24 (57)	
TNM stage			0.537
I / II	48 (48)	52 (52)	
III / IV	24 (43)	32 (57)	
Histological type			0.006
Adenocarcinoma	49 (53)	43 (47)	
Squamous cell carcinoma	12(28)	31 (72)	
Differentiated degree			0.881
Poorly differentiated	13 (43)	17 (57)	
Moderately differentiated	43 (43)	58 (57)	
Highly differentiated	5 (50)	5 (50)

**Table 3:** Summary data for Cox proportional hazards regression analysis of the effect of clinicopathological characteristics and NCAPG expression on overall survival

subtype	Univariate analysis		Multivariate analysis	
	Hazard ratio(95% CI)	*P* -value	Hazard ratio(95% CI)	*P* -value
Gender				
Female	1		1	
Male	2.048 (1.167–3.594)	0.012	1.587 (0.888–2.836)	0.119
Age				
< 60	1			
≥ 60	1.150 (0.679–1.947)	0.603		
TNM Stage				
I / II	1		1	
III / IV	3.033 (1.784–5.155)	< 0.0001	2.770 (1.620–4.738)	< 0.0001
Tumor stage				
pT1	1			
pT2	2.145 (0.889–5.172)	0.089		
pT3	3.436 (1.301–9.077)	0.013		
Node metastasis				
pN0	1			
pN1 / pN2	1.114 (0.579–2.141)	0.747		
Histological type				
Adenocarcinoma	1			
Squamous cell carcinoma Differentiated degree	1.424 (0.772–2.625)	0.258		
Poorly differentiated	1			
Moderately differentiated	1.093 (0.539–2.215)	0.806		
Highly differentiated NCAPG expression	0.816 (0.255–2.610)	0.732		
Negative	1		1	
Positive	2.353 (1.296–4.272)	0.005	2.022 (1.1.06–3.696)	0.022

*CI* confidence interval

### Depletion of *NCAPG* inhibits proliferation, migration, and invasion of NSCLC cells

**Fig. 3:**
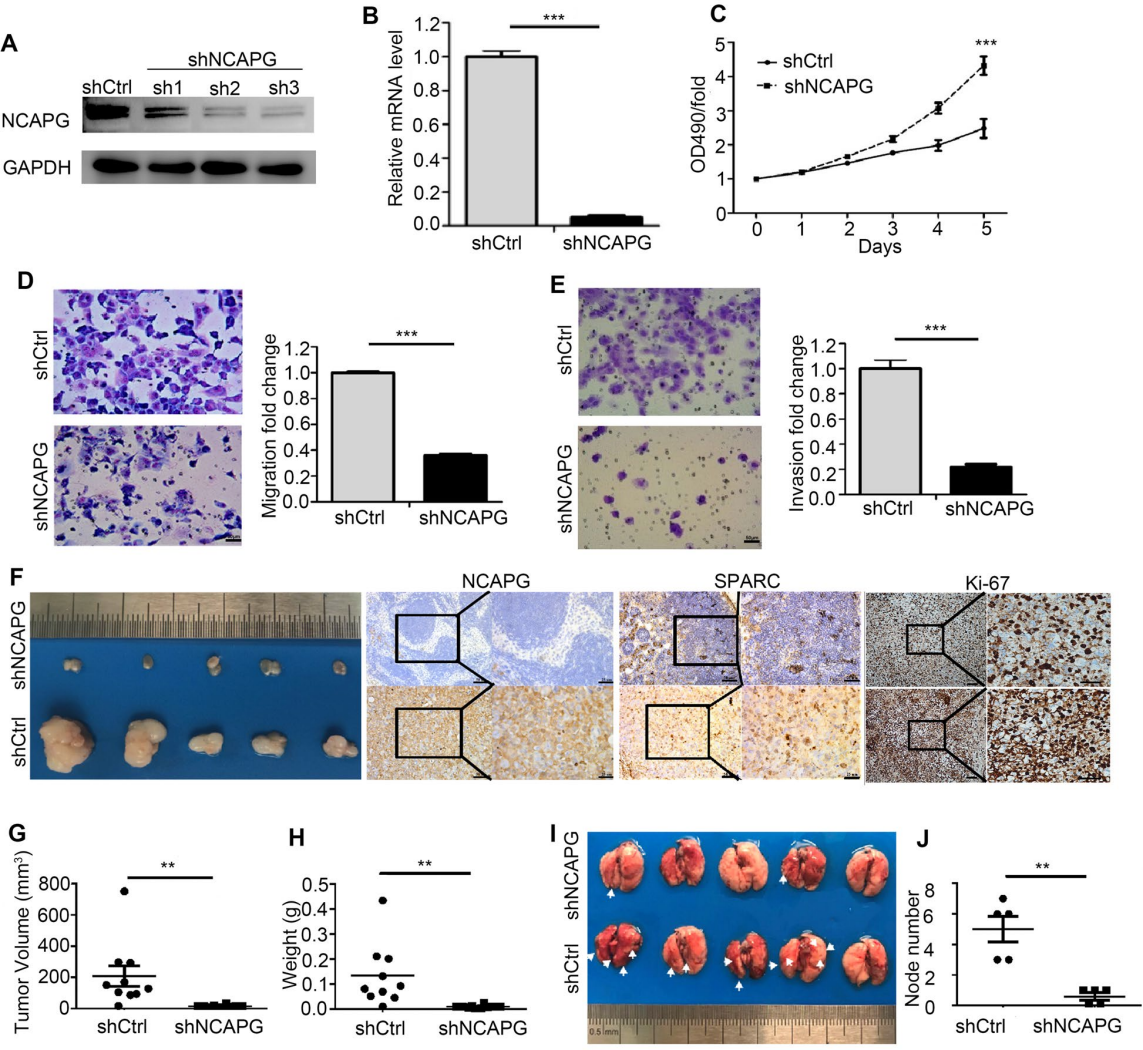
The effects of NCAPG knockdown in vitro and in vivo. **A**, **B** The decreased mRNA and protein expression of *NCAPG* in A549 cells following *NCAPG* knockdown were analyzed using RT-qPCR and Western blot. **C-E** The inhibited abilities of proliferation (**C**), migration (**D**), and invasion (**E**) of A549 cells after *NCAPG* knockdown were shown**.** (*** *p* < 0.001). **F** Tumors dissected from subcutaneous axilla of mice injected with LV-shNCAPG H1299 cells and LV-shCtrl H1299 cells, and the expression of NCAPG, SPARC, and Ki-67 of tumors was tested using immunohistochemistry. **G**, **H** Tumor volumes (**G**) and tumor weights (**H**) of xenografts in LV-shNCAPG and LV-shCtrl group. **I** The photograph of pulmonary tumors formed by injecting LV-shNCAPG or LV-shCtrl H1299 cells into caudal vein. **J** The number of metastatic lung tumors in LV-shNCAPG and LV-shCtrl group

A549 cells transfected with either a control shRNA or shRNAs specifically against NCAPG were detected by Western blot, and the NCAPG inhibitor shRNA3 was the most effective inhibitor of NCAPG and was chosen to treat NSCLC cells (Fig. 3A). Both mRNA and protein expression levels of NCAPG were significantly decreased following NCAPG knockdown in A549 (Fig. 3B, *p* < 0.001) and H1299 cells (Fig. 2S E, F, *p <* 0.05). In addition, *NCAPG* knockdown significantly inhibited cell proliferation, migration, and invasion (Fig. 3C-E, *p <* 0.001; Fig. 2S G-I, *p <* 0.0001). Furthermore, we also have used shRNA2 to transfect NSCLC cells, and achieved similar results that NCAPG knockdown inhibited cell proliferation, migration, and invasion (data not shown).

To explore whether *NCAPG* could influence NSCLC tumor growth in vivo, we performed xenograft experiments on BALB/c nude mice by subcutaneous injection of H1299 cells transfected with either LV-shNCAPG or LV-shCtrl. Immunohistochemistry of xenograft tumor tissues showed that Ki-67 expression was reduced in the LV-shNCAPG tumors (Fig. 3F). These results showed that *NCAPG* knockdown significantly suppressed tumor growth, and that volume and weight of the LV-shNCAPG xenografts were notably decreased compared with that of the LV-shCtrl tumors (Fig. 3F-H, *p <* 0.01). These data were independently confirmed using A549 xenografts transfected with LV-shNCAPG plasmid (Fig. S2 A-D, *p <* 0.05).

In addition, SPARC, a protein regulating the epithelial-mesenchymal-paracrine signaling [17], was also significantly suppressed (Fig. 3F). We suspect that *NCAPG* might play a role in tumor metastasis. To assess the relationship between *NCAPG* and NSCLC tumor metastasis *in vivo*, we established a pulmonary metastasis tumor model in nonobese diabetic/severe (NOD/SCID) mice, and observed that silencing NCAPG in H1299 cells suppressed lung metastases (Fig. 3I, J), indicating that the metastasis ability of tumor is significantly inhibited following *NCAPG* knockdown. However, whether NCAPG promotes NSCLC tumor metastasis through SPARC should be further studied.

### *Ncapg* deficiency suppresses urethane-induced lung cancer in vivo

**Fig. 4:**
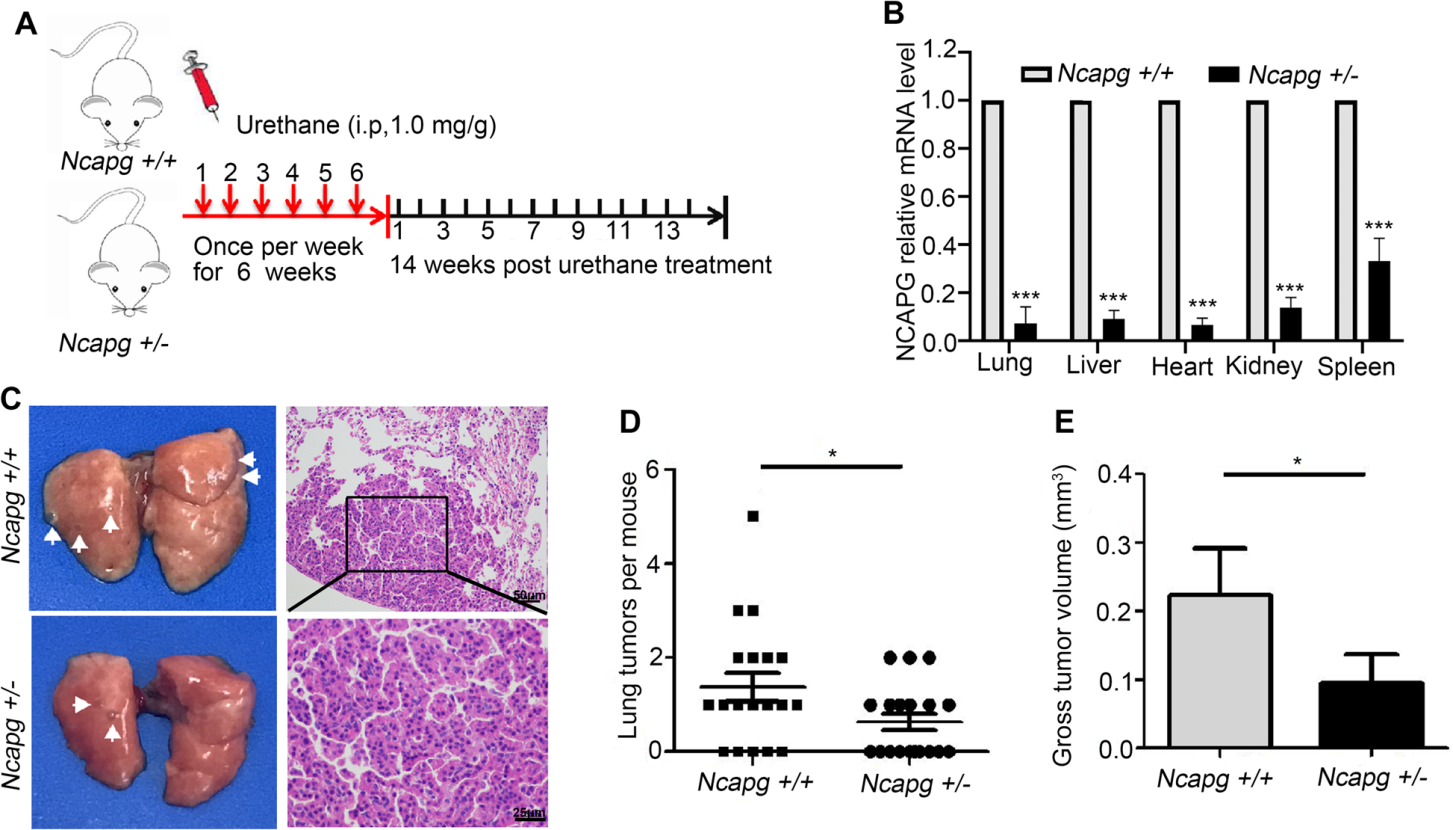
Urethane-induced lung tumor in *Ncapg*^+/+^ and *Ncapg*^+/−^ mice. **A** The strategy of spontaneous lung tumor induced by urethane. **B** *NCAPG* mRNA expression analysis of the main organs in *Ncapg*^+/+^ and *Ncapg*^+/−^ mice. **C** The photograph of urethane-induced lung tumor and representative images of hematoxylin-eosin staining in *Ncapg*^+/+^ and *Ncapg*^+/−^ mice. **D**, **E** Number (**D**) and Volume (**E**) of lung tumors induced by urethane in *Ncapg*^+/+^ and *Ncapg*^+/−^mice. (* *p* < 0.05)

To further investigate the role of *Ncapg* in urethane-induced lung cancer, urethane was administered to *Ncapg*-deficient mice. We generated *Ncapg* gene knockout constructs and crossed heterozygous targeted mice to generate *Ncapg*^−/−^ mice. Due to the embryonic lethality after *Ncapg* knockout (Fig. S3 A, B), only *Ncapg*^+/−^ and *Ncapg*^+/+^ mice could be detected using PCR and agarose gel electrophoresis to test mouse embryonic fibroblasts (MEFs) at embryonic day 9 (Fig. S3 C, D). It has established that consistently repeated urethane exposure results in pulmonary adenoma and adenocarcinoma formation [18]. Therefore, *Ncapg*^+/−^ and wide type C57BL/6 mice were all injected intraperitoneally with urethane to induce lung tumor (Fig. 4A). We confirmed that the mRNA expression of NCAPG in organs of *Ncapg*^+/−^ mouse was attenuated (Fig. 4B). The results indicated that the number of tumor nodules formed in *Ncapg*^*+/−*^ mouse lung tissues was significantly less than that of wide type group (Fig. 4C-E). These data indicate that *NCAPG* plays a significant role in lung tumorigenesis.

### LGALS1 interacts with NCAPG to mediate tumor progression in NSCLC cells

**Fig. 5:**
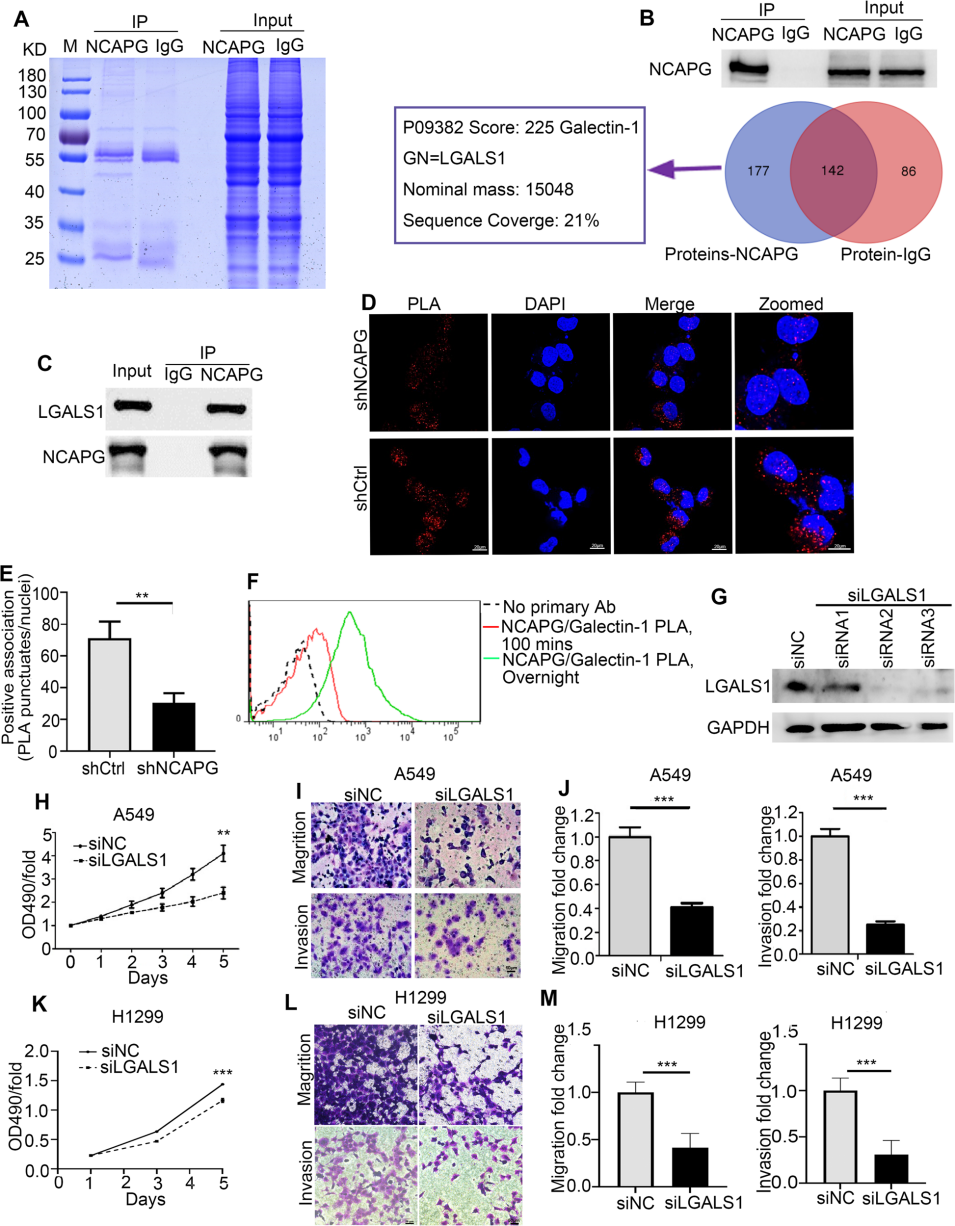
LGALS1 was identified as an important NCAPG-interacting protein. **A** The SDS-PAGE image with Coomassie Blue Staining before mass spectrometry identified NCAPG-interacting proteins in A549 cells. **B** The highest score of NCAPG-interacting protein network was measured with biological function enrichment analysis. **C** LGALS1 was co-precipitated by NCAPG in A549 cells. **D** For each panel, the images from left to right showed PLA signal in A549 cells (red), cell nuclei stained by DAPI (blue), and overlays of the two images. Scale bar 20 μm. **E** The histogram displays the relative fluorescent value of PLA punctuates and nuclei. **F** PLA signal of A549 cells for different conditions by flow cytometer. **G** Western blot analysis for LGALS1 protein after LGALS1 knockdown in A549 cells. **H-M** The abilities of proliferation, migration, and invasion were decreased after LGALS1-knockdown in H1299 and A549 cells. Scale bar 50 μm. (** *p* < 0.01, *** *p* < 0.001)

To elucidate the mechanism by which NCAPG promotes cell proliferation, migration, and invasion in NSCLC, we precipitated and analyzed NCAPG-interacting proteins in A549 cells using Co-Immunoprecipitation (Co-IP) and Liquid Chromatography-Mass Spectrometry (LC-MS), respectively (Fig. 5A). NCAPG-interacting protein networks were scored based on their correlation with DNA replication, recombination, repair, and metabolism functions. LGALS1 (Galectin-1), a β-galactoside binding mammalian lectin, is overexpressed in NSCLC and is associated with cancer progression and immune disorders. We used INGENUITY® database analysis and functional enrichment analysis to identify that LGALS1 was co-precipitated with NCAPG in A549 cells (Fig. 5B, C). Furthermore, we detected a clear positive signal showing the interaction of NCAPG and LGALS1 in A549 cells by PLA coupled with immunofluorescence assays (Fig. 5D, E) and flow cytometer (Fig. 5F). Similarly, the effect of LGALS1 knockdown also inhibited cell proliferation, migration, and invasion in A549 and H1299 cells (Fig. 5G-M).

**Fig. 6:**
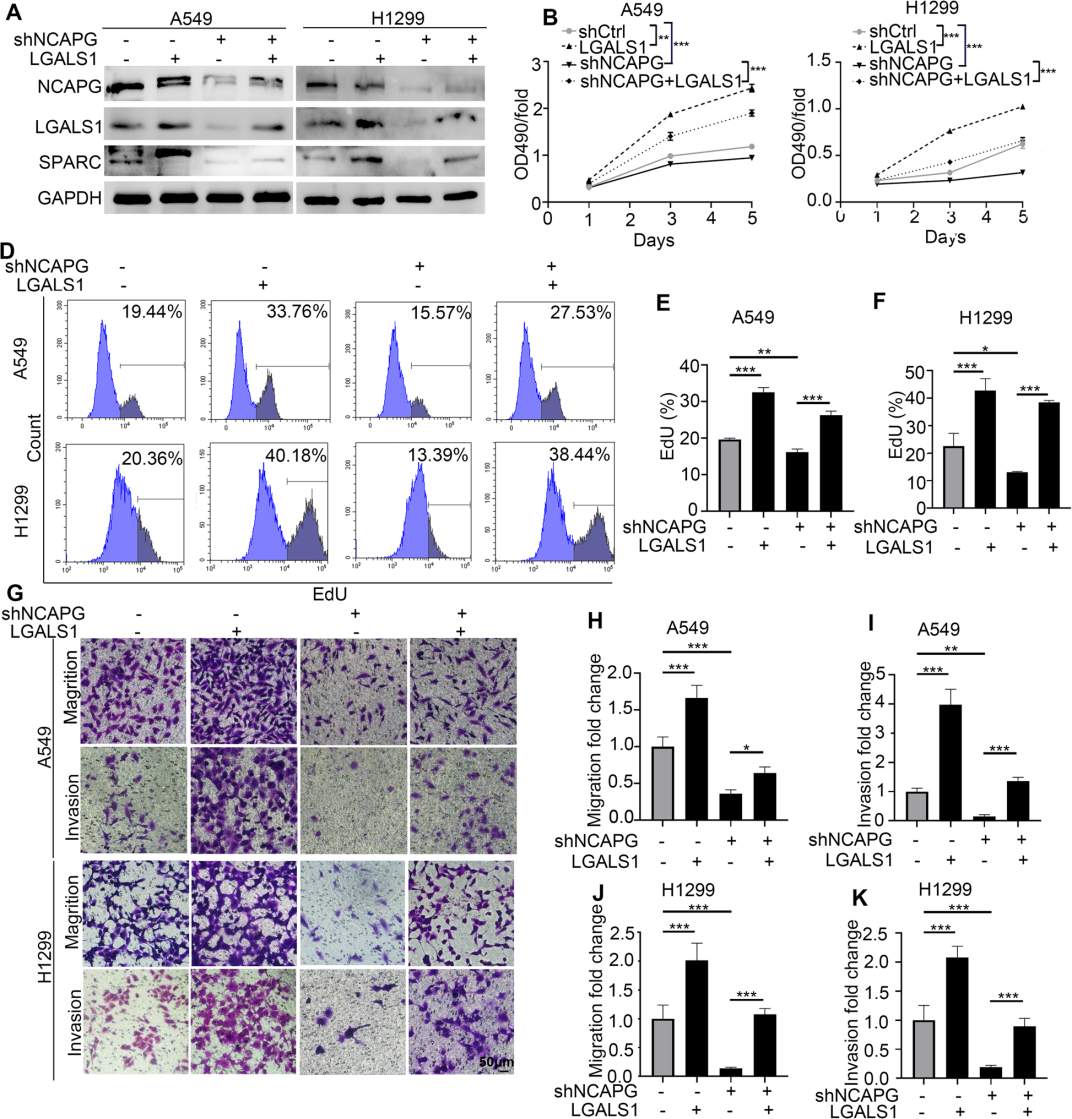
The proliferation and metastasis of NSCLC cells depended on NCAPG/LGALS1/SPARC axis. **A** Western blot was performed in A549 and H1299 cells with NCAPG knockdown and/or LGALS1 overexpression. **B**, **C** CCK8 analysis was used to detect the proliferation of A549 and H1299 cells. **D-F** The proliferation of A549 and H1299 cells was tested by flow cytometer. **G-K** The abilities of migration and invasion were measured through transwell analysis with NCAPG knockdown and/or LGALS1 overexpression. Scale bar 50 μm. (** *p* < 0.01, *** *p* < 0.001)

To confirm whether NCAPG induced NSCLC cells proliferation and metastasis through LGALS1, Western blot were performed to show that NCAPG inhibition remarkably suppressed LGALS1 and SPARC protein levels, while downregulation of SPARC was reversed in LGALS1 overexpression cells (Fig. 6A). MTT, flow cytometry, and transwell analysis were used to detect the proliferation, migration, and invasion of H1299 and A549 cells with NCAPG reduction and/or LGALS1 forced overexpression (Fig. 6B-K). These data showed the functions of proliferation, migration, and invasion in NSCLC cells might partially depend on NCAPG/LGALS1/SPARC axis.

## Discussion

*NCAPG* has been demonstrated to regulate DNA location on chromosomes during mitotic chromosome condensation regulated by the condensin I complex in mitosis process, as a potential candidate target for medicine development. This study discovered that NCAPG expression was negatively associated with NSCLC survival and played a major role in the initiation and progression of NSCLC *in vitro* and *in vivo*. Our study suggests the important role of *NCAPG* in NSCLC and its potential value in diagnosis and prognosis, suggesting that NCAPG represent a promising target for NSCLC treatment. It is critical to identify novel biomarkers and targets for improving NSCLC patients’ clinical outcomes. Using comprehensive analyses of NSCLC cases from the TCGA and the GEO databases, we found that NCAPG was significantly overexpressed in NSCLC tumor tissues and negatively correlated with overall patient survival. Consistent with our findings, previous studies suggest that NCAPG functions as oncogene in cancer cells [9], whose overexpression could promote epithelial-mesenchymal transition, proliferation, and apoptosis suppression in tumor cells [19, 20] . We next investigated the underlying mechanism of NCAPG-mediated proliferation and metastasis in NSCLC cells. Our data revealed that NCAPG interacts with LGALS1 protein resulting in increased cell proliferation, invasion, and migration in vitro and in vivo. The galectin family members of glycan-binding proteins are known for their role in regulating cancer development and progression [21]. LGALS1, a glycan-binding protein, regulates tumor cell proliferation, invasion, and metastasis in pancreatic ductal adenocarcinoma [22]. As a result of our findings, we suggest that NCAPG interacts with LGALS1 to promote proliferation, invasion, and migration in NSCLC cells. Since SPARC overexpression enhances tumor-initiated permeability and vascular leakiness, which finally induces lung metastasis [17], and our study found that NCAPG knockdown could suppress SPARC expression. We speculate that NCAPG might interact with LGALS1 to upregulate SPARC, leading to activate EMT signaling and augment tumor-derived vascular permeability.

With the rapid development of NSCLC treatments over the last two decades, a widespread use has been limited by the fact that targeted therapies require specific genetic background and resistance often occurs. Hence, development of new therapeutic targets is needed. Urethane can combine with DNA to form DNA etheno adducts and is used to artificially develop pulmonary adenomas in neonatal mice [23]. We found that *NCAPG* played a significant role in lung tumorigenesis of a urethane-induced murine lung cancer model. Since NCAPG has been proposed as therapeutic target to overcome trastuzumab resistance in breast cancer [10], our results indicate that NCAPG is not only an important oncogenic driver but may also serve as a new target for treating NSCLC. Future work is warranted to elucidate the mechanisms by which *NCAPG* knockout induces embryonic lethality.

In conclusion, *NCAPG* is required for tumorigenesis and progression of NSCLC. *NCAPG* represents a promising biomarker for NSCLC early diagnosis, prognosis prediction, and drug development. Inhibition of the NCAPG/LGALS1 axis may be a new strategy for NSCLC treatment.

## Supplementary Information

**Additional file 1.**

## Data Availability

Not applicable.

## Abbreviations

ACN: Acrylonitrile Acn
ALK: Anaplastic Lymphoma Kinase
ATCC: American Type Culture Collection
BRAF: B-Rapidly Accelerated Fibrosarcoma
CI: Confidence Interval
Co-IP: Co-Immunoprecipitation
EDTA: Ethylene Diamine Tetraacetic Acid
EGFR: Epidermal Growth Factor Receptor
DMEM: Dulbecco’s Modified Eagle Medium
FBS: Fetal Bovine Serum
FC: Fold Change
FDR: False Discovery Rate
GO: Gene Ontology
HRP: Horseradish Peroxidase
IHC: Immunohistochemistry
KEGG: Kyoto Encyclopedia of Genes and Genomes
KM: KaplanMeier
KRAS: Kirsten Rat Sarcoma Viral Oncogene
LC-MS: Liquid Chromatography-Mass Spectrometry
MEFs: Mouse Embryonic Fibroblasts
MET: MNNG HOS Transforming Gene
NCAPG: Non-SMC Condensin I Complex Subunit G
NOD/SCID: Non-Obese Diabetes/Server Combined Immune-Deficiency
NSCLC: Non-Small Cell Lung Cancer
ROS: Reactive Oxygen Species
RPMI: Roswell Park Memorial Institute Medium
RT-qPCR: Realtime Quantitative PCR
SDS-PAGE: Sodium Dodecyl Sulfate Polyacrylamide Gel Electrophoresis
siRNA: Small Interfering RNA
shRNA: Short Hairpin Interfering RNA
TCGA: The Cancer Genome Atlas
TFA: Trifluoroacetic Acid
TNM: Tumor-node-metastasis
WB: Western Blot
