# Correction: NCAPG promotes the oncogenesis and progression of non-small cell lung cancer cells through upregulating LGALS1 expression

**DOI:** 10.1186/s12943-022-01689-4

**Published:** 2022-12-14

**Authors:** Huanhuan Sun, Hong Zhang, Yan Yan, Yushi Li, Gang Che, Cuiling Zhou, Christophe Nicot, Haiqing Ma

**Affiliations:** 1 Medical Research Center, Guangdong Provincial People’s Hospital, Guangdong Academy of Medical Sciences, 106 Zhongshan Er Rd, Guangzhou, 510080 Guangdong China; 2 Department of Oncology, The Fifth Affiliated Hospital, Sun Yat-sen University, Zhuhai, China; 3 Department of Oncology, Guangdong Cardiovascular Institute, Guangdong Provincial People’s Hospital, Guangdong Academy of Medical Sciences, Guangzhou, China; 4 Department of Pathology and Laboratory Medicine, University of Kansas Medical Center, 3901 Rainbow Boulevard, Kansas City, KS 66160 USA

**Correction: Mol Cancer 21, 55 (2022)**

**https://doi.org/10.1186/s12943-022-01533-9**

In the originally published version of this article [1], for Fig. 2I (the bottom row on the right), the label of the 4th lane should be ‘H1975’ after checking the original records, and the correct label was replaced.

**Fig. 2:**
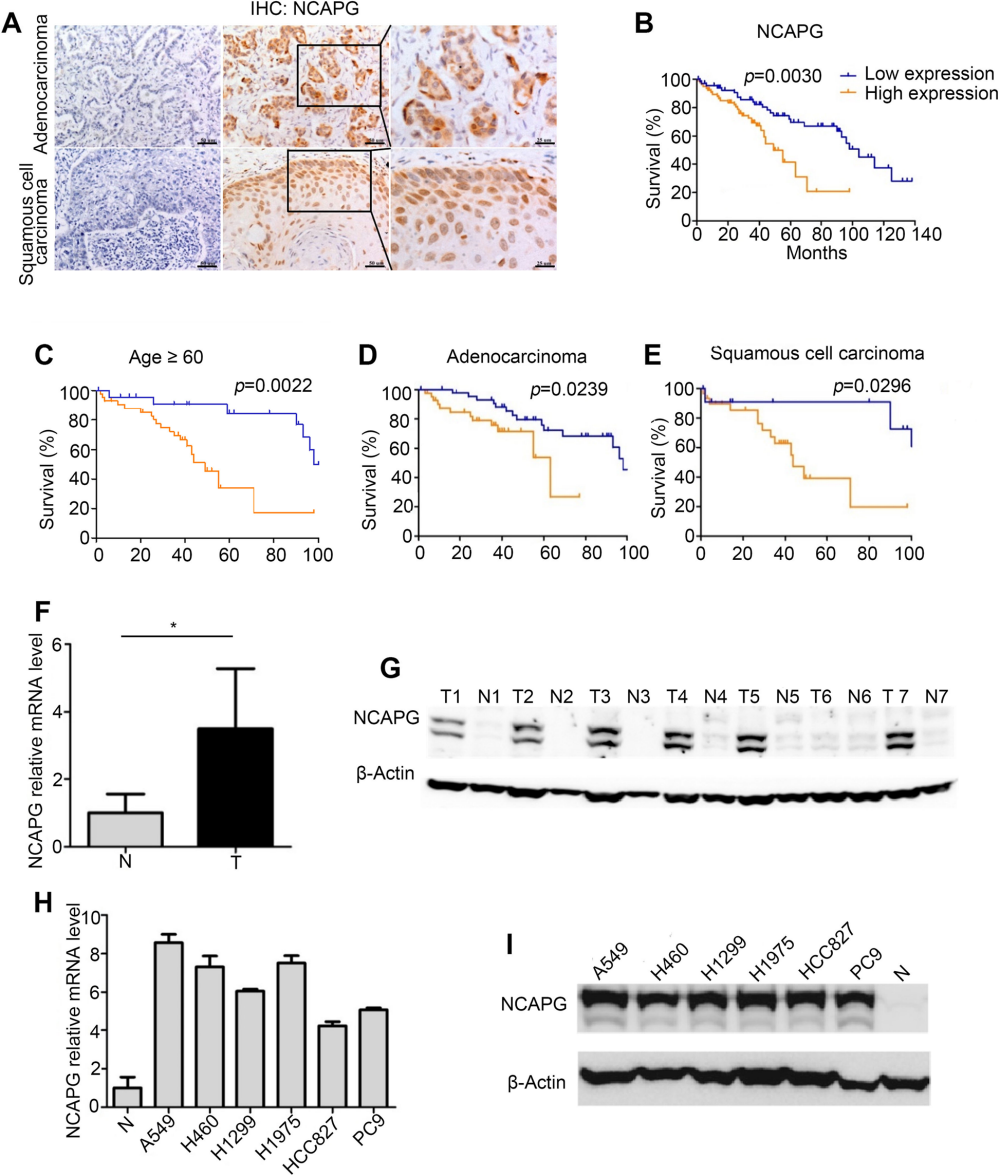
Overexpression of *NCAPG* in NSCLC tissues and cell lines. **A** NCAPG expression in NSCLC tumor tissues by IHC. Left: negative; middle & right: positive; top: adenocarcinoma; bottom: squamous cell carcinoma. **B** The relationship between NCAPG expression and survival in NSCLC patients. **C**-**E** This negative correlation between NCAPG expression and survival was identified in elderly patients (≥ 60 years old) (*n* = 84, *p* = 0.0022), adenocarcinoma (*n* = 92, *p* = 0.0239), and squamous cell carcinoma (*n* = 43, *p* = 0.0296). **F**, **G** *NCAPG* mRNA (**F**) and protein expression (**G**) in NSCLC tumor tissues (*n* =21) (T: tumor tissues, N: matched adjacent normal tissues). **H**, **I** *NCAPG* mRNA (N: Average *NCAPG* mRNA expression in adjacent normal tissues (*n* = 21)) and protein (N: Representative protein level of *NCAPG* in adjacent normal tissues) expression in NSCLC cell lines

In the originally published version of this article [1], Fig. 4D (the bottom row in the middle), was used incorrectly after checking the original records, and the correct picture was replaced.

**Fig. 4:**
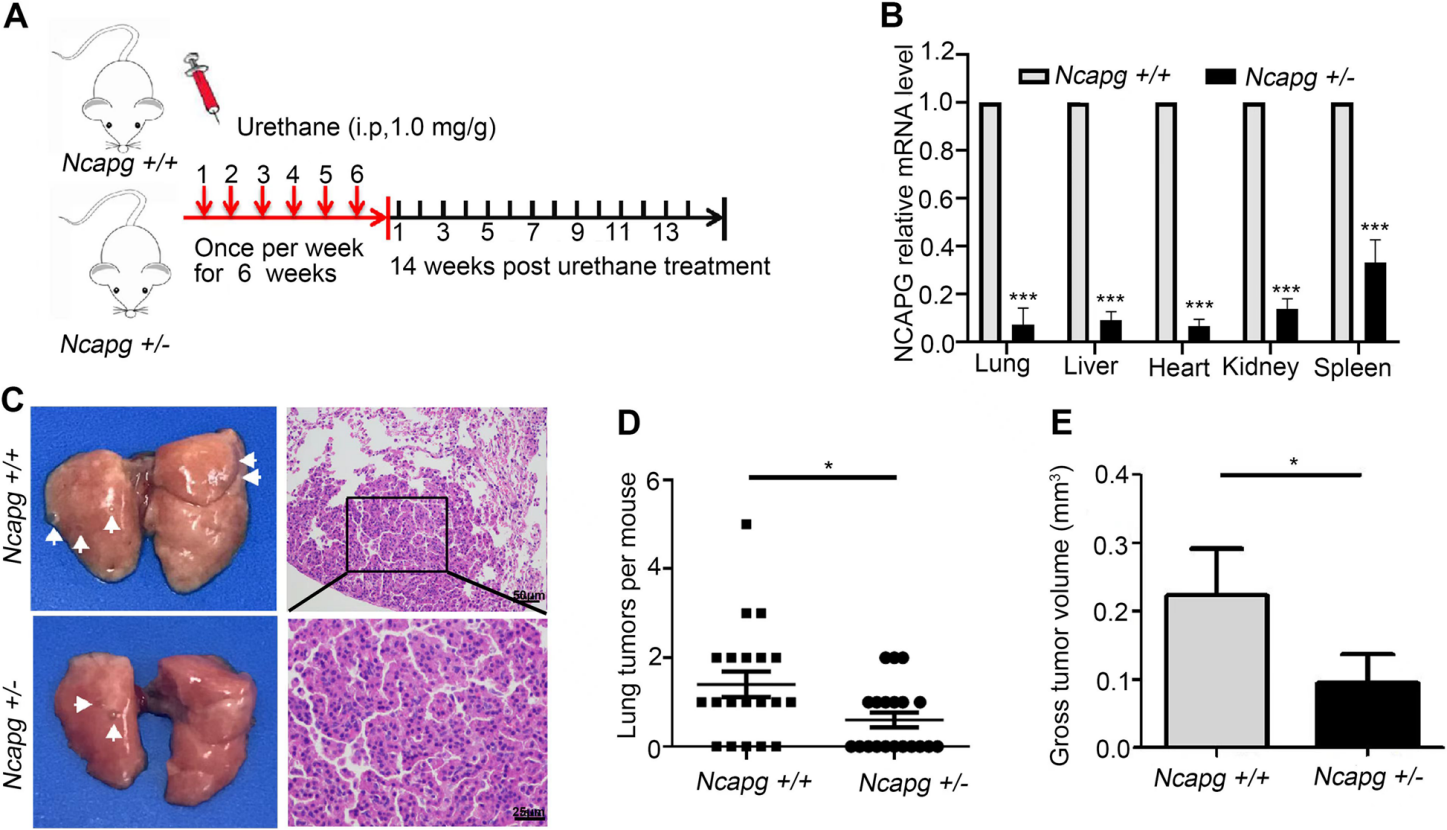
Urethane-induced lung tumor in *Ncapg*^+/+^ and *Ncapg*^+/−^ mice. **A** The strategy of spontaneous lung tumor induced by urethane. **B** *NCAPG* mRNA expression analysis of the main organs in *Ncapg*^+/+^ and *Ncapg*^+/−^ mice. **C** The photograph of urethane-induced lung tumor and representative images of hematoxylin-eosin staining in *Ncapg*^+/+^ and *Ncapg*^+/−^ mice. **D**, **E** Number (**D**) and Volume (**E**) of lung tumors induced by urethane in *Ncapg*^+/+^ and *Ncapg*^+/−^ mice. (* *p* < 0.05)
