# Supplementary material for: NCAPG promotes the oncogenesis and progression of non-small cell lung cancer cells through upregulating LGALS1 expression
Source: Mol Cancer. 2022 Feb 18;21:55. doi: 10.1186/s12943-022-01533-9 (PMC8855584; doi:10.1186/s12943-022-01533-9)
Supplement: Supplementary file 1 — Additional file 1. [file 12943_2022_1533_MOESM1_ESM.docx]

**NCAPG promotes the oncogenesis and progression of non-small cell lung cancer cells through upregulating LGALS1 expression**

**Huanhuan Sun^1,2#^, Hong Zhang^3#^, Yan Yan^2#^, Yushi Li^2^, Gang Che^2^,** **Cuiling Zhou^2^, Christophe Nicot^4^*** **and Haiqing Ma^1,2,3^***

1 Medical Research Center, Guangdong Provincial People's Hospital, Guangdong Academy of Medical Sciences, Guangzhou, China

2 Department of Oncology, The Fifth Affiliated Hospital, Sun Yat-sen University, Zhuhai, China

3 Department of Oncology, Guangdong Cardiovascular Institute, Guangdong Provincial People's Hospital, Guangdong Academy of Medical Sciences, Guangzhou, China

4 Department of Pathology and Laboratory Medicine, University of Kansas Medical Center, 3901 Rainbow Boulevard, Kansas City, KS, 66160, USA.

^#^ These authors contributed equally to this work.

* Corresponding Author: Christophe Nicot, Department of Pathology and Laboratory Medicine, University of Kansas Medical Center, 3901 Rainbow Boulevard, Kansas City, KS, 66160, USA. cnicot@kumc.edu. Haiqing Ma, Guangdong Provincial People's Hospital, Guangdong Academy of Medical Sciences, 106 Zhongshan Er Rd, Guangzhou, Guangdong 510080, China. Phone: +86-(20)-81884713-80511; E-mail: mahaiqing@gdph.org.cn

**Materials and methods**

**RNA-sequencing and analysis**

Total RNA was extracted from the samples by Trizol reagent (Invitrogen) separately. The cDNA libraries were constructed for each RNA sample using the TruSeq Stranded mRNA Library Prep Kit (Illumina, Inc.) according to the manufacturer’s instructions. Then the clean reads were aligned to human genome (GRCh38, NCBI) using the Hisat2 [[1](#_ENREF_1)]. HTseq was used to get gene counts, while RPKM method was used to determine the gene expression [[2](#_ENREF_2)]. DESeq algorithm was applied to filter the differentially expressed genes [[3](#_ENREF_3)], after the significant analysis, P-value and FDR analysis were subjected to the following criteria: i) Fold Change > 2 or < 0.5; ii) p-value < 0.05, FDR < 0.05 [[4](#_ENREF_4)]. Gene ontology (GO) analysis was performed to elucidate the biological implications of the differentially expressed genes in the experiment [[5](#_ENREF_5)]. Pathway analysis was used to find out the significant pathway of the differentially expressed genes according to KEGG database [[6](#_ENREF_6)]. The significant GO-Term in GO Analysis based on the up and down differentially expressed genes was selected to construct the GO-Tree to summarize the function affected in the experiment [[7](#_ENREF_7)]. The genes in enriched biological pathway were picked and Cytoscape was used for graphical representation of pathways [[8](#_ENREF_8)].

**Western blot analysis**

Western blot (WB) analysis was performed to detect the NCAPG and LGALS1 protein expression. To perform WB analysis, the membranes were first incubated with anti-NCAPG (at a 1:500 dilution), anti-LGALS1 (at a 1:500 dilution), or anti-GAPDH (at a 1:5,000 dilution). After washing, the membranes were then incubated with a secondary HRP-conjugated antibody (at a 1:10,000 dilution). Details of WB analysis were described previously [[9](#_ENREF_9)].

**RNA extraction and RT-qPCR analysis**

RNA extraction and RT-qPCR analysis were performed as previously described[[9](#_ENREF_9)]. The RT-PCR primer pairs used were as follows: *NCAPG* forward, 5’-GCTCAGAGAATGCTCCTTCAACAA-3’ and reverse, 5’-CTTCAGAGAACCGTAACCAGCCTTG-3’; *LGALS1* forward, 5’-GAGGTGGCTCCTGACGCTAAGA-3’ and reverse, 5’-TCCTTGCTGTTGCACACGATGG-3’; *GAPDH* forward, 5’-CAAGGTCATCCATGACAACTTTG-3’ and reverse, 5’-GTCCACCACCCTGTTGCTGTAG-3’. Primer sequences of 13 up-regulated genes are provided in Table S4.

**Immunohistochemistry**

The procedures were described previously [[9](#_ENREF_9)]. The primary antibody was mouse anti-NCAPG (H00023397-M01), Novus Biologicals, at a 1:800 dilution.

**[Lentiviral shRNA packaging and short interfering RNAs (siRNAs) transfection](C:/Users/18924/AppData/Local/youdao/dict/Application/7.5.2.0/resultui/dict/../dict/result.html?keyword=Lentiviral%20RNAi%20expression%20vector&lang=en)**

The procedures of plasmid constructs were described previously [[9](#_ENREF_9)]. Plasmids of the lentiviruses (LV)-shControl (shCtrl) and LV-shNCAPG (shNCAPG) were prepared by Shanghai Genechem Co. (Shanghai, China). The LV-shNCAPG plasmid was transfected into cells to silence the *NCAPG* gene and the LV-shCtrl plasmid was transfected into cells as the control group. Control (Ctrl) and LGALS1-targeted siRNA (si LGALS1) were purchased from GeneCopoeia (Guangzhou, China). These two plasmids were transfected into targeting cells using Lipofectamine 3000 (Invitrogen, Carlsbad, CA, USA) according to the manufacturer’s instructions, respectively.

LGALS1-targeted siRNA sequences:

siRNA1 F: CUGACGGUGACUUCAAGAUTT

siRNA1 R: AUCUUGAAGUCACCGUCAGTT

siRNA1 F: CGCUAAGAGCUUCGUGCUGAA

siRNA1 R: UUCAGCACGAAGCUCUUAGCG

siRNA1 F: CCAGCAACCUGAAUCUCAATT

siRNA1 R: UUGAGAUUCAGGUUGCUGGTT

**Cell proliferation assay**

Cell proliferation was detected by MTT assay, which was performed as described previously [[10](#_ENREF_10)].

**Cell invasion and migration assays**

Cell invasion and migration were evaluated by using the transwell assay. After 24 h serum-starved culture, H1299 and A549 cells (25000 to 50000) were suspended in 100 μL of serum-free medium and loaded into 8.0 μm pore polycarbonate membrane inserts (Corning, NY 14831 USA), in duplicates. Cell migrated towards the lower chamber containing 600 μL of medium with 10% FBS for 24 h. The migrated cells were fixated with 4% paraformaldehyde and stained with 1% crystal violet blue. Non-migrating cells were removed with cotton swab and migrated cells were counted in five random microscopic fields. Invasion assay was performed as migration assay with Matrigel™ (Corning) which was spread on the transwell insert before cells migration [[11](#_ENREF_11)].


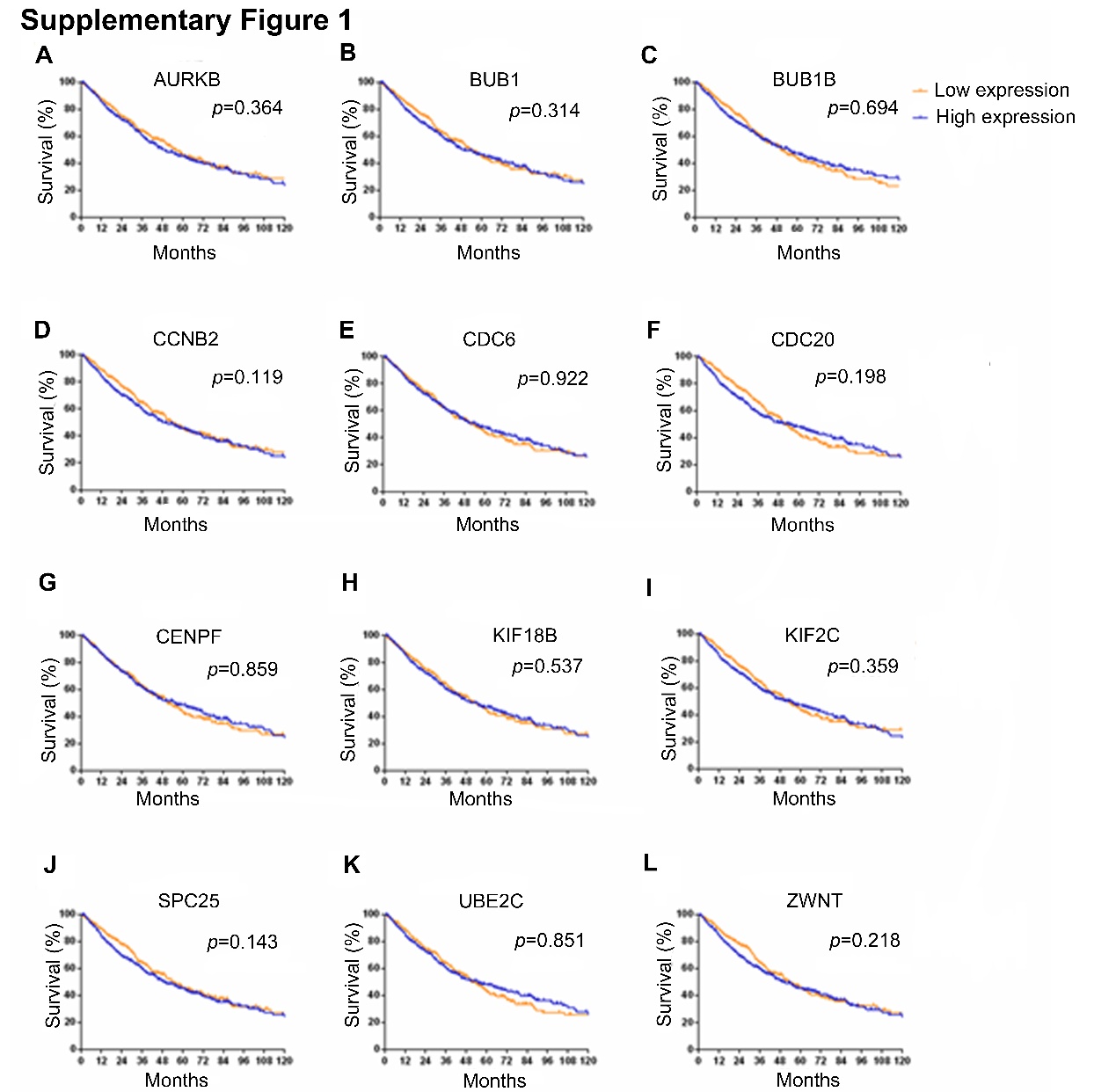


**Fig. S1. Correlation between the expression of 12 up-regulated genes and NSCLC survival.**

**
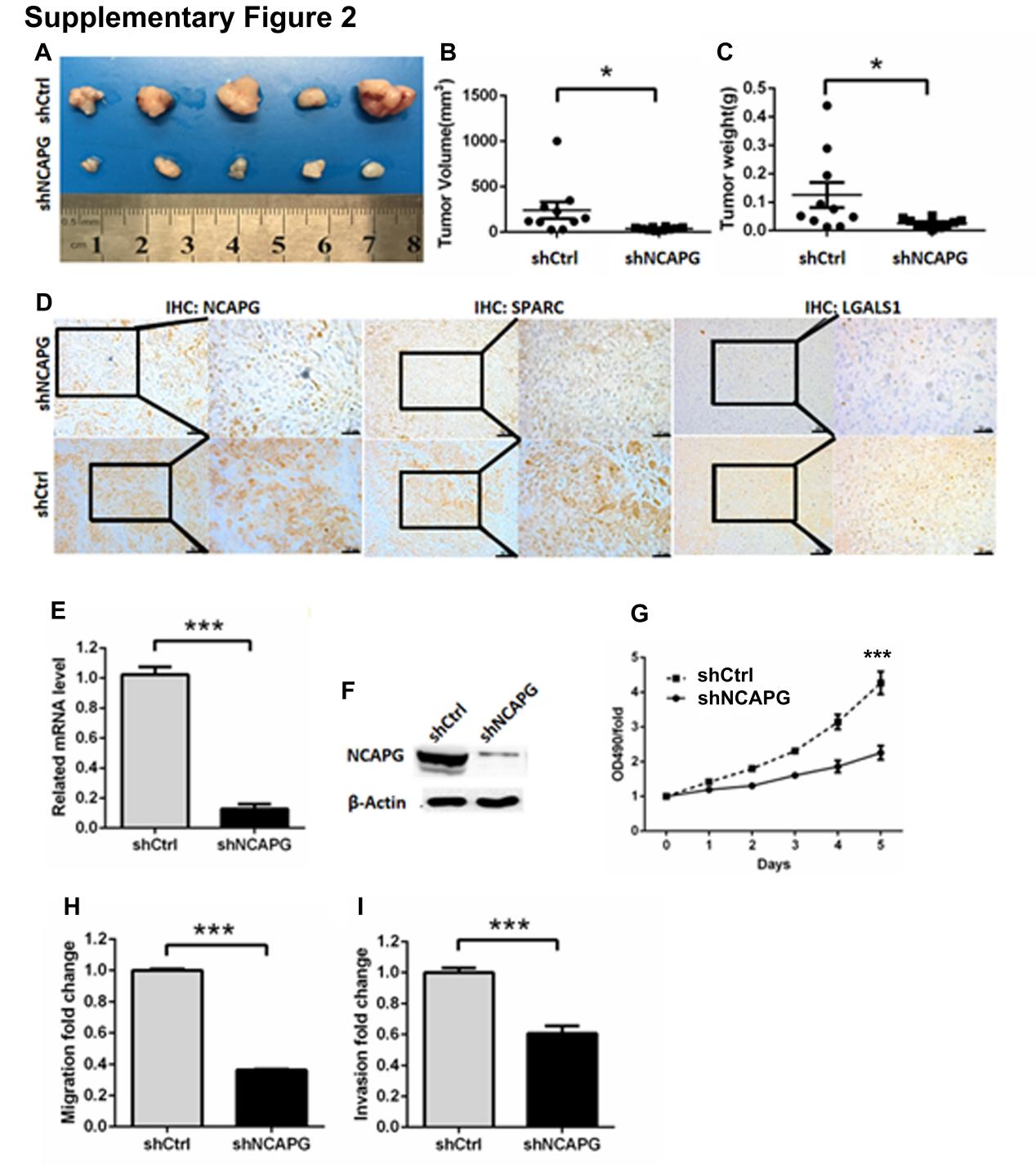
Fig. S2. The effects of *NCAPG* knockdown on cell proliferation, migration and invasion of A549 and H1299 cells. A**. Tumors were dissected from subcutaneous axilla of mice injected with LV-shNCAPG A549 cells and LV-shCtrl A549 cells. **B-C**. Tumor volumes (B) and tumor weights (C) of xenografts. **D**. The expression of NCAPG, SPARC and LGALS1 of the tumors were showed by IHC. **E, F.** The mRNA (**E**) and protein (**F**) expression of *NCAPG* in H1299 cells with *NCAPG* knockdown. **G-I**. The proliferation (**G**), migration (**H**) and invasion (**I**) abilities of H1299 cells with *NCAPG* knockdown.

**
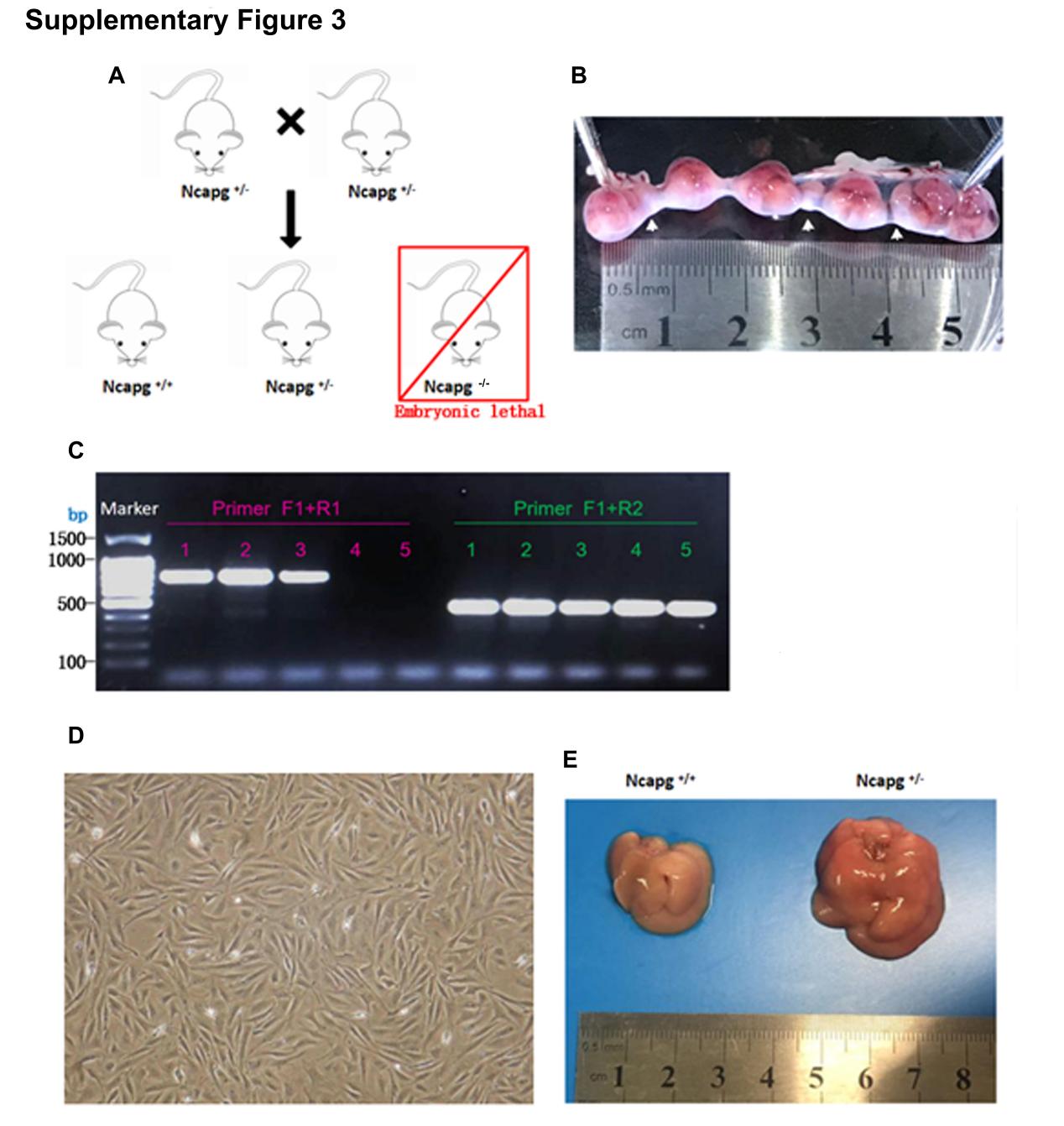
**

**Fig. S3. Non *Ncapg*^-/-^ mice was born during the intercross of *Ncapg*^+/-^ mice. A**. Intercrossing strategy was used to generate *Ncapg*^-/-^ mice. **B**. The embryos of homozygous mice (white arrows) at embryonic day 9 were atrophic and necrotic. **C**. Genotype identification of *Ncapg* transgenic mice. Homozygotes (760 bp), heterozygotes (760 bp/439 bp), wild type allele (439 bp). **D.** The fibroblasts were derived from *Ncapg*^+/-^ embryos. **E.** liver of *Ncapg*^+/+^ and *Ncapg*^+/-^ mice.


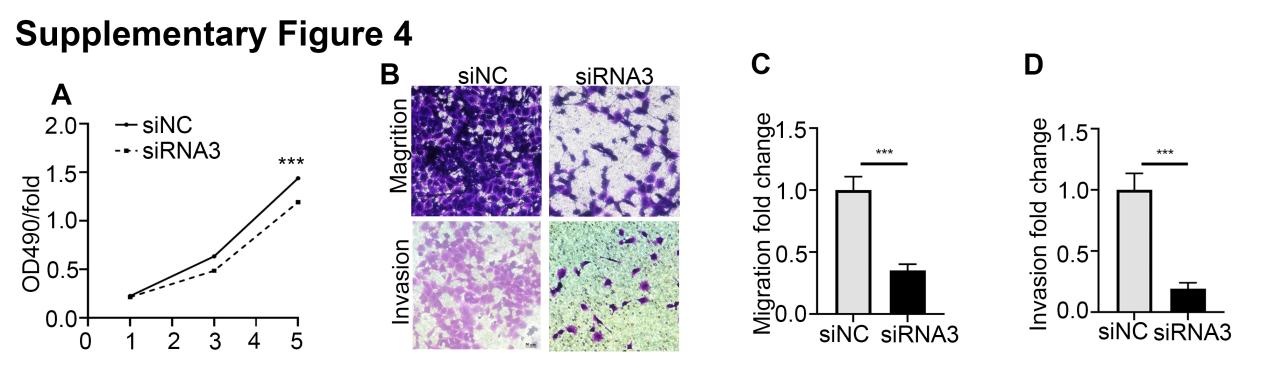


**Fig. S4. The effects of another *LGALS1* siRNA on cell proliferation, migration and invasion of H1299 cells. A**-**D**. The proliferation (**A**), migration (**B, C**) and invasion (**B, D**) abilities of H1299 cells with *LGALS1* knockdown.

**Table S1.** The clinicopathological features of clinical NSCLC patients (n=156).

| Characteristic | Total (N = 156) | | |  |
| --- | --- | --- | --- | --- |
|  | Number of patients | % | |  |
| Gender |  |  |  | |
| Male | 90 | 58 |  |  |
| Female | 66 | 42 |  |  |
| Age |  |  |  |  |
| <60 | 84 | 54 |  |  |
| ≥60  Tumor stage  pT1  pT2  pT3  pT4  Node metastasis  pN0  pN1  pN2 | 72  31  72  25  4  87  14  28 | 46  20  46  16  3  56  9  18 |  |  |
| TNM stage |  |  |  |  |
| I  II  III  IV  Histological type  Adenocarcinoma  Squamous cell carcinoma  Differentiated degree  Poorly differentiated  Moderately differentiated  Highly differentiated  NCAPG  Negative  Positive | 61  39  43  13  92  43  30  101  10  72  84 | 39  25  28  8  59  28  19  65  6  46  54 |  |  |

**Table S2.** Genotype results of mouse embryonic fibroblasts (MEFs) from offspring mice embryos at embryonic day 9, 10, 12 and 14.

| \| MEF  Genotype \| Wide Type  (*Ncapg*^+/+^) \| Heterozygote  (*Ncapg*^+/-^) \| Knock Out  (*Ncapg*^-/-^) \| Total Embryos Number \| \| --- \| --- \| --- \| --- \| --- \| \| E14d  E12d  E10d  E9d \| 1  5  2  0 \| 9  5  4  12 \| 0  0  0  0 \| 10  10  6  12 \| |
| --- | --- | --- | --- | --- | --- | --- | --- | --- | --- | --- |

**Table S3.** Primer sequences of 13 up-regulated genes.

| **Genes** | **Forward** | **Reverse** |
| --- | --- | --- |
| **AURKB** | \| GAGCAGCGAACAGCCACGAT \|  \|  \| \| --- \| --- \| --- \| | \| CAGACCAGCCGAAGTCAGCAAT \|  \|  \| \| --- \| --- \| --- \| |
| **BUB1** | \| GGAAGTGCCTCATGCTGAAGAGTT \|  \|  \|  \| \| --- \| --- \| --- \| --- \| | \| TGTGGAATGGTGTAGACGCAAGTT \|  \|  \| \| --- \| --- \| --- \| |
| **BUB1B** | \| CCAGCAGACAGCTTGTGGCACTATC \|  \|  \|  \| \| --- \| --- \| --- \| --- \| | \| GCAACCGAGGCAGAAGAACCAGAG \|  \|  \| \| --- \| --- \| --- \| |
| **CCNB2** | \| GCTGGTACAAGTCCACTCCAAGTT \|  \|  \| \| --- \| --- \| --- \| | \| AAGCCAAGAGCAGAGCAGTAATCC \|  \|  \| \| --- \| --- \| --- \| |
| **CDC6** | \| CCAGGCACAGGCTACAATCAGTT \|  \|  \| \| --- \| --- \| --- \| | \| TTACACGAGGAGAACAGGTTACGG \|  \|  \| \| --- \| --- \| --- \| |
| **CDC20** | \| AAGACCTGCCGTTACATTCCTTCC \|  \|  \| \| --- \| --- \| --- \| | \| ACATTCCCAGAACTCCAATCCACAA \|  \|  \|  \| \| --- \| --- \| --- \| --- \| |
| **CENPF** | \| CCAGACTCTTCCACAAGCCACCAT \|  \|  \| \| --- \| --- \| --- \| | \| TGCTGCCATGAGAACACAGATGATG \|  \|  \|  \| \| --- \| --- \| --- \| --- \| |
| **KIF2C** | \| GCTGGAGGAGAAGGCTATGGAAGAG \|  \|  \|  \| \| --- \| --- \| --- \| --- \| | \| TAGTCTGGCTGCTCGGTCATCTCA \|  \|  \| \| --- \| --- \| --- \| |
| **KIF18B** | \| AATCCCTGTGCCGTCTCCTCTCT \|  \|  \| \| --- \| --- \| --- \| | \| CGCTTGGTCTCCTCCTCTTCTTCTC \|  \|  \|  \| \| --- \| --- \| --- \| --- \| |
| **NCAPG** | \| GCTCAGAGAGTAATGCCCTTCAACAA \|  \|  \|  \| \| --- \| --- \| --- \| --- \| | \| CTTCAGAGAACCGTAACCAGCCTTG \|  \|  \|  \| \| --- \| --- \| --- \| --- \| |
| **SPC25** | \| TGGCGGGACTAAGAGATACCTAC \|  \|  \| \| --- \| --- \| --- \| | \| TCTCAACCATTCGTTCTTCTTCCTT \|  \|  \|  \| \| --- \| --- \| --- \| --- \| |
| **UBE2C** | \| ATGTCTGGCGATAAAGGGATTTCT \|  \|  \| \| --- \| --- \| --- \| | \| CCTCAGGTCTTCATATACTGTTCCA \|  \|  \|  \| \| --- \| --- \| --- \| --- \| |
| **ZWINT** | \| GGCATCTTGGAACCTGTAGGC \|  \|  \| \| --- \| --- \| --- \| | \| TGTCGGCTCGTGTCTTCAGAA \|  \|  \| \| --- \| --- \| --- \| |

**References:**

1. Kim D, Langmead B, Salzberg SL: **HISAT: a fast spliced aligner with low memory requirements.** *Nat Methods* 2015, **12:**357-360.

2. Anders S, Pyl PT, Huber W: **HTSeq--a Python framework to work with high-throughput sequencing data.** *Bioinformatics* 2015, **31:**166-169.

3. Anders S, Huber W: **Differential expression analysis for sequence count data.** *Genome Biol* 2010, **11:**R106.

4. Benjamini Y, Drai D, Elmer G, Kafkafi N, Golani I: **Controlling the false discovery rate in behavior genetics research.** *Behav Brain Res* 2001, **125:**279-284.

5. Ashburner M, Ball CA, Blake JA, Botstein D, Butler H, Cherry JM, Davis AP, Dolinski K, Dwight SS, Eppig JT, et al: **Gene ontology: tool for the unification of biology. The Gene Ontology Consortium.** *Nat Genet* 2000, **25:**25-29.

6. Draghici S, Khatri P, Tarca AL, Amin K, Done A, Voichita C, Georgescu C, Romero R: **A systems biology approach for pathway level analysis.** *Genome Res* 2007, **17:**1537-1545.

7. Zhang B, Schmoyer D, Kirov S, Snoddy J: **GOTree Machine (GOTM): a web-based platform for interpreting sets of interesting genes using Gene Ontology hierarchies.** *BMC Bioinformatics* 2004, **5:**16.

8. Shannon P, Markiel A, Ozier O, Baliga NS, Wang JT, Ramage D, Amin N, Schwikowski B, Ideker T: **Cytoscape: a software environment for integrated models of biomolecular interaction networks.** *Genome Res* 2003, **13:**2498-2504.

9. Ma H, Liang X, Chen Y, Pan K, Sun J, Wang H, Wang Q, Li Y, Zhao J, Li J, et al: **Decreased expression of BATF2 is associated with a poor prognosis in hepatocellular carcinoma.** *Int J Cancer* 2011, **128:**771-777.

10. Ma H, Weng D, Chen Y, Huang W, Pan K, Wang H, Sun J, Wang Q, Zhou Z, Wang H, Xia J: **Extensive analysis of D7S486 in primary gastric cancer supports TESTIN as a candidate tumor suppressor gene.** *Mol Cancer* 2010, **9:**190.

11. Tan DS, Haaland B, Gan JM, Tham SC, Sinha I, Tan EH, Lim KH, Takano A, Krisna SS, Thu MM, et al: **Bosutinib inhibits migration and invasion via ACK1 in KRAS mutant non-small cell lung cancer.** *Mol Cancer* 2014, **13:**13.
